# Deep Cavernosal Melanoma Without Clinically Evident Mucosal or Cutaneous Involvement: A Case Report

**DOI:** 10.1155/criu/4792867

**Published:** 2026-07-22

**Authors:** M. J. Iglesias, M. Bogliaccini, L. Mouro, C. Villa

**Affiliations:** ^1^ Department of Urology, Hospital Maciel, Montevideo, Uruguay

**Keywords:** case report, corpus cavernosum, immunotherapy, penile melanoma, urology

## Abstract

**Background:**

Penile melanoma is an extremely rare malignancy, representing less than 0.1% of all melanomas. It most commonly arises from the glans, foreskin, or urethral meatus. Deep cavernosal involvement without clinically evident cutaneous, mucosal, or urethral disease is an unusual presentation and poses significant diagnostic and therapeutic challenges.

**Case Presentation:**

A 23‐year‐old male presented with progressive penile induration and pain. MRI revealed a lesion predominantly involving the corpus cavernosum, with no clinically evident cutaneous, mucosal, or urethral involvement. An excisional biopsy established the diagnosis of malignant melanoma; however, the initial specimen showed tumor involvement of the resection margin and did not include epidermal sampling. Systemic staging with whole‐body CT and PET‐CT showed no evidence of metastatic disease. The patient subsequently underwent partial penectomy with bilateral sentinel lymph node dissection, showing negative sentinel lymph nodes and free final surgical margins. One year later, 18F‐FDG PET‐CT demonstrated nodal and osseous metastatic disease, with hypermetabolic lymphadenopathies in the distal retroperitoneal, left iliac, and left inguinal regions, osseous lesions in the sternal manubrium, and a new right upper‐lobe pulmonary micronodule. The patient received immunotherapy with a limited clinical response.

**Conclusion:**

This case describes an unusual presentation of melanoma predominantly involving the corpus cavernosum without clinically evident cutaneous, mucosal, or urethral involvement. The absence of epidermal sampling prevents definitive histological exclusion of a regressed or deeply invasive mucosal/cutaneous primary lesion. This report highlights the aggressive behavior of deeply located penile melanoma and the need for careful clinicopathological correlation and individualized multidisciplinary management.

## 1. Introduction

Penile melanoma is an extremely rare malignancy, accounting for less than 0.1% of all melanomas and approximately 2% of penile tumors [1, 2]. The most commonly affected sites are the glans, foreskin, and urethral meatus [1, 3]. Prognosis is generally poor, with reported 5‐year survival rates ranging from 10% to 30%, largely due to delayed diagnosis [4, 5].

Several case reports and small series have been published, including early experiences from Spain [6–10] and larger European and international cohorts [1, 2, 11]. However, melanoma with predominant corpus cavernosum involvement in the absence of clinically evident cutaneous, mucosal, or urethral disease is exceptionally uncommon. Such presentations pose diagnostic challenges, particularly in distinguishing a truly primary deep penile melanoma from a deeply invasive or regressed mucosal/cutaneous primary lesion. We present a rare case of melanoma predominantly involving the corpus cavernosum and discuss the clinicopathological findings, diagnostic limitations, therapeutic approach, and clinical evolution.

## 2. Case Presentation

A 23‐year‐old male presented with progressive penile induration and pain of 6 months’ duration. Physical examination revealed a palpable mass along the right corpus cavernosum, without clinical involvement of the glans, foreskin, or urethral meatus. No ulceration or pigmentation of the penile skin or mucosa was observed.

Magnetic resonance imaging (MRI) demonstrated a 3.5‐cm lesion predominantly located within the corpus cavernosum, with low signal intensity on T1‐weighted images and heterogeneous enhancement. Cystoscopy showed no evidence of urethral involvement.

**Figure 1:**
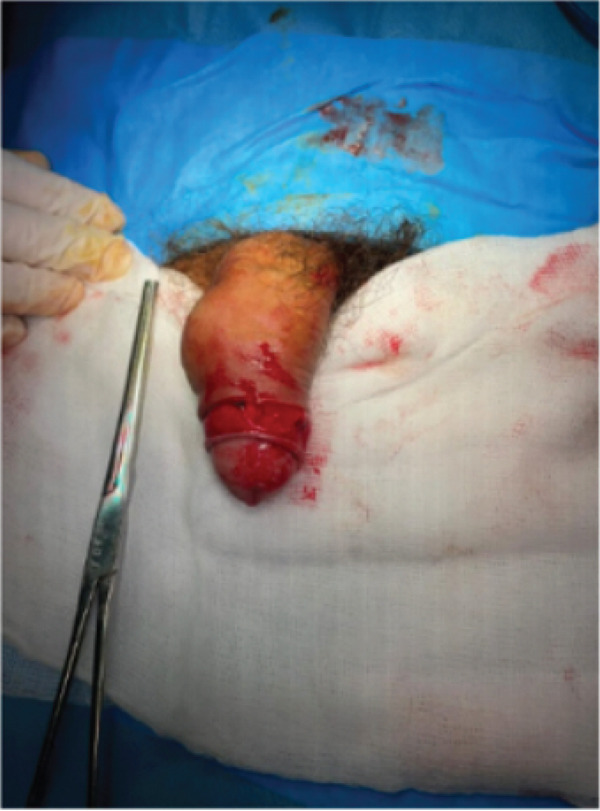
Intraoperative view showing deformation of the penile shaft contour caused by the underlying cavernosal lesion before tumor dissection.

**Figure 2:**
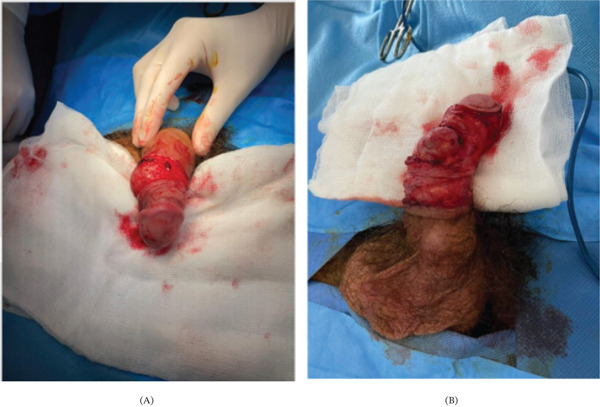
Intraoperative appearance of the penile lesion. (A) Initial exposure showing a nodular deformity of the penile shaft. (B) Gross appearance after penile shaft exposure, demonstrating the deep location of the lesion.

**Figure 3:**
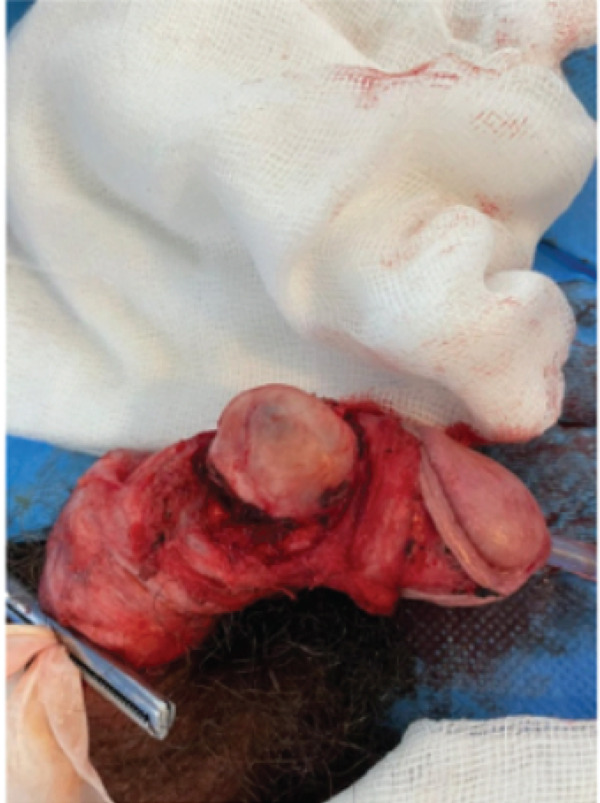
Intraoperative view after surgical exposure of the lesion, showing a well‐defined nodular tumor involving the region of the corpus cavernosum.

**Figure 4:**
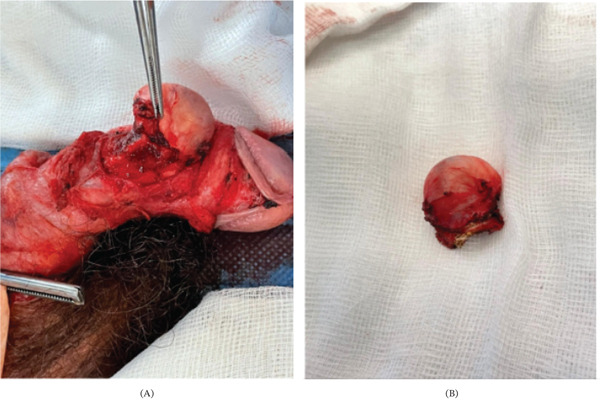
Surgical excision of the cavernosal lesion. (A) Tumor dissection from the surrounding cavernosal tissue. (B) Gross surgical specimen sent for histopathological examination.

An excisional biopsy was performed, providing the initial histopathological diagnosis. Intraoperative examination showed deformation of the penile shaft contour caused by the underlying cavernosal lesion (Figure 1). Further surgical exposure demonstrated the nodular deformity and deep location of the lesion within the penile shaft (Figure 2). After complete exposure, a well‐defined nodular tumor involving the corpus cavernosum was identified (Figure 3). The lesion was dissected from the surrounding cavernosal tissue and excised for histopathological examination (Figure 4). The specimen showed a malignant melanocytic proliferation composed of spindle to epithelioid cells, focally with clear cell change, with high mitotic activity and perineurial invasion. Tumor involvement of the resection margin was reported. Immunohistochemistry demonstrated diffuse positivity for SOX10, S100, HMB‐45, and Melan‐A, while cathepsin K and SMA were negative. Molecular analysis by fluorescence in situ hybridization was negative for ATF1, CRTC1, and EWSR1 rearrangements, supporting the diagnosis of melanoma and helping to exclude clear cell sarcoma and other melanocytic mimickers.

Importantly, the epidermis was not sampled in the initial specimen. Therefore, although no cutaneous, mucosal, urethral, or distant primary lesion was identified clinically, endoscopically, or radiologically, a regressed or deeply invasive cutaneous/mucosal primary lesion could not be definitively excluded on histological grounds. Given the predominant deep cavernosal involvement and the absence of clinically evident cutaneous or mucosal disease, conventional cutaneous melanoma staging based on Clark level was not considered applicable.

Following the histopathological diagnosis of melanoma, a complete dermatological and dermatoscopic examination was performed to exclude a primary cutaneous lesion. Upper and lower gastrointestinal endoscopic evaluation, including esophagogastroduodenoscopy and colonoscopy, was also performed to rule out a digestive primary site. Systemic staging was subsequently performed with whole‐body CT and PET‐CT, neither of which demonstrated metastatic disease.

The patient subsequently underwent partial penectomy with bilateral sentinel lymph node dissection. Histopathological examination of the partial penectomy specimen confirmed malignant melanoma involving the corpus cavernosum, with free final surgical margins. Sentinel lymph nodes were negative.

Postoperative recovery was uneventful. The case was discussed in a multidisciplinary tumor board. Given the absence of clinically detectable residual disease after definitive surgery, free final surgical margins, negative sentinel lymph nodes, and no evidence of distant metastasis on initial staging, adjuvant systemic therapy was not recommended at that time.

After definitive local treatment, the patient showed no evidence of local residual tumor or systemic disease. However, during follow‐up 1 year later, 18F‐FDG PET‐CT demonstrated disease progression, with multiple hypermetabolic lymphadenopathies involving the distal retroperitoneal region, left iliac chain, and left inguinal region. These included a lateroaortic lymph node before the aortic bifurcation measuring 7 mm (SUVmax 3.4), left iliac lymphadenopathies measuring up to 9 mm (SUVmax 4.4), a left external iliac nodular lesion measuring 28 mm with peripheral uptake and central hypometabolism (SUVmax 2.9), and left inguinal lymphadenopathies measuring up to 13 mm (SUVmax 7.2). A 20‐mm hypermetabolic prepubic subcutaneous nodular lesion lateral to the penile shaft was also identified (SUVmax 5.2), together with a mildly hypermetabolic focal lesion in the distal penile shaft related to the right corpus cavernosum (SUVmax 3.2). Hypermetabolic osseous lesions were detected in the sternal manubrium (SUVmax 3.8). A new 4‐mm micronodule in the apical segment of the right upper pulmonary lobe was also observed, below the metabolic resolution of PET. The patient was subsequently treated with immunotherapy, with limited clinical response.

## 3. Discussion

Penile melanoma most frequently arises from the glans, foreskin, or urethral meatus [1, 2, 6–10]. In the present case, the tumor showed predominant involvement of the corpus cavernosum, without clinically evident involvement of the glans, foreskin, urethral meatus, penile skin, or urethra. This represents an unusual anatomical presentation and highlights the diagnostic challenges of deeply located penile melanocytic tumors.

A major limitation of this case is the lack of histological sampling of the overlying epidermis in the initial specimen. Consequently, a regressed superficial component or a deeply invasive mucosal/cutaneous melanoma with secondary cavernosal involvement cannot be definitively excluded. Another limitation is the lack of representative histopathological images. Because of the diagnostic complexity of the lesion, the case was referred for external expert pathology consultation in the Czech Republic, including additional immunohistochemical and molecular analyses, and the original slides or representative digital images were not available to the authors for inclusion. The interpretation of predominant cavernosal involvement was, therefore, based on the combination of clinical examination, dermatoscopic assessment, cystoscopy, MRI, systemic staging with CT and PET‐CT, and pathological findings. For this reason, the case has been reframed as a melanoma predominantly involving the corpus cavernosum without clinically evident mucosal, cutaneous, or urethral involvement, rather than as a definitively proven primary cavernosal melanoma.

Because no epidermal component was available for histological assessment and the tumor was deeply located within the cavernosal tissue, conventional cutaneous melanoma staging based on Clark level was not considered applicable. In this context, systemic staging with whole‐body CT and PET‐CT was essential, and no evidence of metastatic disease was detected at initial diagnosis.

Diagnosis of penile melanoma is often delayed, as it may mimic benign penile conditions or other malignancies [3, 4]. In the present case, the initial differential diagnosis included clear cell sarcoma of soft tissue and other melanocytic tumors. Imaging, biopsy, immunohistochemistry, and molecular analysis were, therefore, essential for accurate characterization. Diffuse positivity for SOX10, S100, HMB‐45, and Melan‐A supported the diagnosis of melanoma, while negative ATF1, CRTC1, and EWSR1 rearrangements helped exclude clear cell sarcoma and related mimickers.

Therapeutic management of penile melanoma is generally extrapolated from cutaneous and mucosal melanoma guidelines [5, 12, 13]. Surgical excision with negative margins remains the cornerstone of local treatment. In this case, the initial excisional biopsy showed tumor involvement of the resection margin, and definitive local treatment was achieved with partial penectomy, which demonstrated free final surgical margins. Sentinel lymph nodes were negative.

The presence of high‐risk pathological features, including deep cavernosal involvement, perineurial invasion, and initial margin involvement, underscores the aggressive biological potential of this tumor. The case was discussed in a multidisciplinary tumor board. Given the absence of clinically detectable residual disease after definitive surgery, free final surgical margins, negative sentinel lymph nodes, and no evidence of distant metastasis on initial staging, adjuvant systemic therapy was not recommended at that time. Nevertheless, the subsequent development of nodal and osseous metastatic disease within 1 year highlights the need for close follow‐up and suggests that the optimal role of adjuvant systemic therapy in such rare presentations remains uncertain.

Given the rich vascularization of the corpus cavernosum and the initial margin‐positive excisional biopsy, early hematogenous dissemination related either to the aggressive tumor biology or, theoretically, to surgical manipulation is biologically plausible. However, because initial staging with whole‐body CT and PET‐CT showed no evidence of metastatic disease, it is not possible to determine whether the subsequent metastases reflected occult micrometastatic disease already present at diagnosis or potential dissemination associated with local surgical manipulation. This uncertainty represents an important limitation of the case.

Systemic therapies, particularly immune checkpoint inhibitors such as nivolumab and ipilimumab, have demonstrated activity in mucosal melanoma [14]. However, evidence regarding their efficacy in penile melanoma remains limited because of the rarity of reported cases. The limited clinical response observed in our patient further illustrates the uncertainty regarding systemic treatment outcomes in this setting and reinforces the need for additional clinical evidence to guide management.

## 4. Conclusion

Penile melanoma is an uncommon malignancy with a poor prognosis, usually involving the glans, foreskin, or urethral meatus. This case describes an unusual presentation of melanoma predominantly involving the corpus cavernosum, without clinically evident cutaneous, mucosal, or urethral involvement at diagnosis. The absence of epidermal sampling prevents definitive histological exclusion of a regressed or deeply invasive mucosal/cutaneous primary lesion. Nevertheless, this case highlights the importance of considering melanoma in the differential diagnosis of atypical deep penile lesions; the role of imaging, immunohistochemistry, and molecular analysis; and the need for individualized multidisciplinary management and close follow‐up.

## Funding

No funding was received for this manuscript.

## Consent

Written informed consent was obtained from the patient for publication of this case report and any accompanying images.

## Conflicts of Interest

The authors declare no conflicts of interest.

## General Statement

*Patient Perspective.* The patient emphasized the importance of seeking early medical evaluation for persistent penile symptoms and agreed to share his clinical course to contribute to medical knowledge about this rare presentation.

## Supporting information


**Supporting Information** Additional supporting information can be found online in the Supporting Information section. File S1: Anonymized pathology and molecular report supporting the diagnosis of melanoma.

## Data Availability

The data that support the findings of this study are available on request from the corresponding author. The data are not publicly available due to privacy or ethical restrictions.
