# Supplementary material for: Deep Cavernosal Melanoma Without Clinically Evident Mucosal or Cutaneous Involvement: A Case Report
Source: Case Rep Urol. 2026 Jul 22;2026:4792867. doi: 10.1155/criu/4792867 (PMC13392520; doi:10.1155/criu/4792867)
Supplement: Supplementary file 1 — Supporting Information Additional supporting information can be found online in the Supporting Information section. File S1: Anonymized pathology and molecular report supporting the diagnosis of melanoma. [file CRIU-2026-4792867-s001.pdf]

Supplementary File 1. Pathology and Molecular Report

Consultation Report

Case: 211740
Patient: ML Cabrera Rodríguez (ID 5010787-8)
Age: 23 years
Specimen: Penile tumor, 3 x 2.2 x 2 cm

Clinical note: Suspicion of clear cell sarcoma of soft tissue (CCS). Differential diagnosis included melanoma. No evidence of melanoma elsewhere.

Histopathology & Immunohistochemistry:
- Malignant tumor with nodular arrangement, composed of spindled to epithelioid cells, focally with clear cell change.
- High mitotic activity and perineurial invasion present.
- No necrosis identified.
- Tumor extends to the resection margin.
- Epidermis not sampled.
- Immunoprofile: diffusely positive for SOX10, S100, HMB45, Melan A; negative for Cathepsin K and SMA.

Differential diagnosis considered:
- Clear cell sarcoma of soft tissue (CCS).
- Melanoma.
- Dermal melanocytic tumor with CRTC1-TRIM11 fusion.

Molecular analysis (FISH):
- ATF1 gene break: Negative.
- CRTC1 gene break: Negative.
- EWSR1 gene break: Negative.

Final Diagnosis:
Melanoma.

Responsible Pathologist:
Michael Michal, M.D., Ph.D.
Bioptická laborato■ s.r.o.
Mikulasske nam. 4, Plzen, Czech Republic
Email: michael.michal@biopticka.cz

Report date: November 2021
